# Alteration of Intestinal Microbiome of *Clostridioides difficile-*Infected Hamsters during the Treatment with Specific Cow Antibodies

**DOI:** 10.3390/antibiotics10060724

**Published:** 2021-06-16

**Authors:** Hans-Jürgen Heidebrecht, Ilias Lagkouvardos, Sandra Reitmeier, Claudia Hengst, Ulrich Kulozik, Michael W. Pfaffl

**Affiliations:** 1 Food- and Bioprocess Engineering, TUM School of Life Science, Technical University of Munich, Weihenstephaner Berg 1, 85354 Freising, Germany; claudia.hengst@tum.de (C.H.); ulrich.kulozik@tum.de (U.K.); 2 ZIEL—Institute for Food & HealthCore Facility Microbiome, Technical University of Munich, Weihenstephaner Berg 3, 85354 Freising, Germany; ilias.lagkouvardos@tum.de (I.L.); sandra.reitmeier@tum.de (S.R.); 3 Animal Physiology and Immunology, TUM School of Life Science, Technical University of Munich, Weihenstephaner Berg 3, 85354 Freising, Germany; michael.pfaffl@wzw.tum.de

**Keywords:** microbiome, *C. difficile*, hamsters, bovine immunoglobulins, 16S rRNA, next generation sequencing

## Abstract

*Clostridioides difficile* infection (CDI) often develops after pretreatment with antibiotics, which can lead to damage of the intestinal microbiome. The approach of this study was to use specific polyclonal antibodies isolated from the milk of immunized cows to treat CDI, in contrast to the standard application of nonspecific antibiotics. To gain a deeper understanding of the role of the microbiome in the treatment of CDI with bovine antibodies, stool and intestinal fluid samples of hamsters were collected in large quantities from various treatments (>400 samples). The results show that the regeneration of the microbiome instantly begins with the start of the antibody treatment, in contrast to the Vancomycin-treated group where the diversity decreased significantly during the treatment duration. All antibody-treated hamsters that survived the initial phase also survived the entire study period. The results also show that the regeneration of the microbiome was not an antibody-induced regeneration, but a natural regeneration that occurred because no microbiota-inactivating substances were administered. In conclusion, the treatment with bovine antibodies is a functional therapy for both the acute treatment and the prevention of recurrence in hamsters and could meet the urgent need for CDI treatment alternatives in humans.

## 1. Introduction

*Clostridioides difficile* infection (CDI) is a leading healthcare-acquired infection characterized by severe diarrhea and high morbidity rates [1]. Risk factors for CDI are exposure to *C. difficile* spores through (a) community sources such as hospitals or long-term care facilities, (b) host factors such as immune status or comorbidities and (c) substances that interfere with the native commensal intestinal microbiome, such as antibiotics, surgery or other drugs [2]. The risk of CDI is six-fold higher within one month following antibiotic treatment [3]. Disruption of the indigenous microbiota creates conditions that allow the germination and further proliferation of *C. difficile* even though the mechanism is not yet fully understood. In its vegetative state, virulent strains of the obligate anaerobic bacteria *C. difficile* produce the toxins A (TcdA) and B (TcdB), that damage the intestinal epithelium, and which ultimately may cause the death of the patient. Paradoxically, CDI which is induced by antibiotic alteration of the native gut flora, is also treated with antibiotics by default (>95% of the cases). The standard antibiotics to fight and cure CDI are Metronidazole, Fidaxomicin and Vancomycin. These antibiotics suppress the growth of vegetative *C. difficile* and, thus, the initial response is typically good. However, the drawback is that these antibiotics are not specific for *C. difficile* but also cause a further destruction of the already damaged gastrointestinal microbiota. Thus, after discontinuation, patients are susceptible for recurrence of CDI due to the germination of resident and resistant spores, or due to reinfection with spores from an environmental source. In consequence 10–30% of the patient suffer from recurrent CDI after initial apparently successful therapy [2], which increases to 50–65% after the second recurrence [4].

The approach of this work is an alternative way to fight and cure CDI by using specific polyclonal antibodies obtained from bovine milk of immunized cows. Applying a cocktail of inactivated antigens in the vaccine (*C. difficile*, TcdA and TcdB), it is possible to simultaneously induce the formation of a mixture of polyclonal antibodies. The effectiveness of this approach for oral CDI treatment was demonstrated in our previous study [5], and before that in animal models such as hamsters [6,7], gnotobiotic piglets [8], mice [9] and human studies [6,10,11]. However, there is a lack of information about how the microbiota actually changes its composition, diversity and richness while treatment with bovine antibodies is ongoing. The only study touching this aspect was published by Sponseller et al. (2015) [8]. However, only stool samples from two hyper bovine colostrum treated pigs were compared with samples from two untreated pigs, so that no deeper or statistical analysis between the groups was made. To gain a deeper understanding of the role of the microbiome in the treatment of CDI with bovine polyclonal antibodies, in our study stool and intestinal fluid samples of the hamsters were collected on a large scale in various treatment groups (>400 samples). High-throughput deep sequencing was performed on an Illumina MiSeq targeting the V3–V4 hypervariable region of the 16S rRNA gene. The objective of this study was to show which treatment revealed a faster and better regeneration of the natural (pretreatment) gastrointestinal microbiome, either the specific antibodies against *C. difficile* or commonly used antibiotics. To extend the understanding of the role of the microbiome in CDI, independent of the applied treatments, different metabolites were quantified and correlated with *C. difficile* spore or vegetative cell numbers and the respective sequencing data.

## 2. Results

### 2.1. Comparison of Treatment Groups

**Figure 1:**
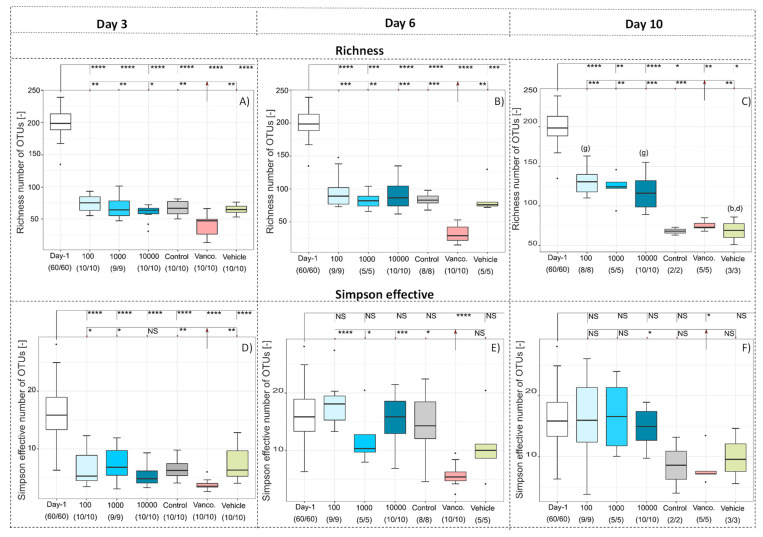
Richness (**A**–**C**) and Simpson effective index (**D**–**F**) during the six different treatments (WPI 10,000, WPI 1000, WPI 100, control WPI, Vancomycin, Vehicle) after 3 (**A**,**D**), 6 (**B**,**E**) and 10 (**C**,**F**) days compared to the 60 values of the hamsters before treatment (day -1). Significance values are *p* < 0.05 = *, *p* < 0.01 = **, *p* < 0.001 = ***, *p* < 0.0001 = ****, NS = not significant. Significant differences between the groups are marked by small letters. The number in parentheses indicates the number of samples and correlates with the number of hamsters surviving at that time.

Figure 1 shows the richness, i.e., the number of OTUs in a sample and the Simpson effective index, of the six different treatment groups on days three, six and 10. It is clear from the richness that around 200 different bacterial species were detected in each stool sample before therapy. The number of species in all groups decreased significantly compared to before the treatment to about 60–80 by treatment day three (*p* < 0.001). The individual significance levels are shown in the graphs. There were no significant differences within the groups, except for the group treated with Vancomycin, where the number was significantly (*p* < 0.05 for WPI 10,000, *p* < 0.01 for all other groups) lower, compared to all other groups. (Figure 1A). The decrease from day one to day three was due to the administration of the susceptibility antibiotic Clindamycin, according to the study design. Analogous to the pretreatment with antibiotics in humans, the antibiotic causes a reduction of intestinal microorganisms, which reduces the competition for *C. difficile* and thus enables the germination of its spores. The survival of the hamsters was WPI 10,000 = 100%; WPI 1000 = 50%; WPI 100 = 80%; Control-WPI = 10%; Vancomycin 10%; vehicle = 0% [5]. The significantly lower number in the treatment group can be explained by the antibiotic treatment, which is known to reduce the number of microbial species and diversity when applied. The increase in the significance level compared to the other treatment groups shows that the difference was even more pronounced after six days. While the number of OTUs in the group treated with antibiotics continued to decrease (*p* < 0.01) the number of species in all other groups increased again (Figure 1B).

By day 10, many hamsters in the control groups died from CDI; therefore, there is a clear difference from the antibody-treated groups (Figure 1C). Richness, however, does not provide any information about the relative diversity of the different OTUs. For a better representation of the community structures, therefore, the Simpson effective index was calculated (Figure 1D,E). The Simpson effective index is a measure of how equally diverse a community is, whereby high numbers stand for high diversity and low numbers for lower diversity. Analogous to the richness, intestinal microbial diversity decreased significantly (*p* < 0.001) in all groups by day three (Figure 1D). However, already from day six onwards (Figure 1E,F), there was no significant difference between the different treatment groups compared to the pretreatment group, except for the group treated with Vancomycin, which showed a significantly lower diversity (*p* < 0.05–0.0001). This means that the intestinal microbiome from day six onwards was equally diverse in all groups as before treatment, except the group treated with Vancomycin. Combining the results of richness and Simpson effective index, the number of bacterial species was not as high as before, probably because reduced occurrence of rare species, but the microbiome regenerated in terms of diversity from day six. There were only minor differences in the regeneration of diversity between the groups treated with polyclonal antibodies and the control groups. Based on the results it can be concluded that the regeneration of the microbiome was probably not an active antibody-induced regeneration. It was rather based on the fact that no microbiota-inactivating substances were administered, which led to natural microbiome regeneration, e.g., by ingestion of food. However, the comparison also revealed that the regeneration of the microorganisms alone was not sufficient for the survival of the hamsters, since almost all hamsters in the control groups died although the diversity of the microbiome increased, as the numbers in brackets show [5]. It should also be noted that the informative value of the microbiome within the control groups decreased over the course of the study due to the high mortality rate of hamsters. Nevertheless, this means that an active and antibody-induced mechanism must have taken place in addition to the regeneration of the microbiome, which caused survival and needs to be investigated further.

**Figure 2:**
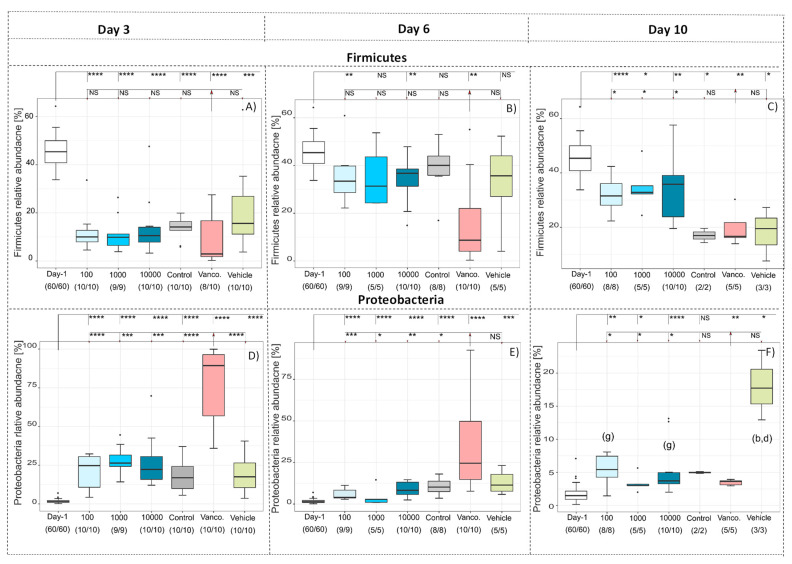
Relative abundance at phylum level of Firmicutes (**A**–**C**), Proteobacteria (**D**–**F**) after 3 (**A**,**D**), 6 (**B**,**E**) and 10 (**C**,**F**) days at indicated treatment compared to the relative abundance before treatment (day -1). Significance compared to before treatment and vancomycin values are *p* < 0.05 = *, *p* < 0.01 = **, *p* < 0.001 = ***, *p* < 0.0001 = ****, NS = not significant. Significant differences between the groups are marked by small letters.

**Figure 3:**
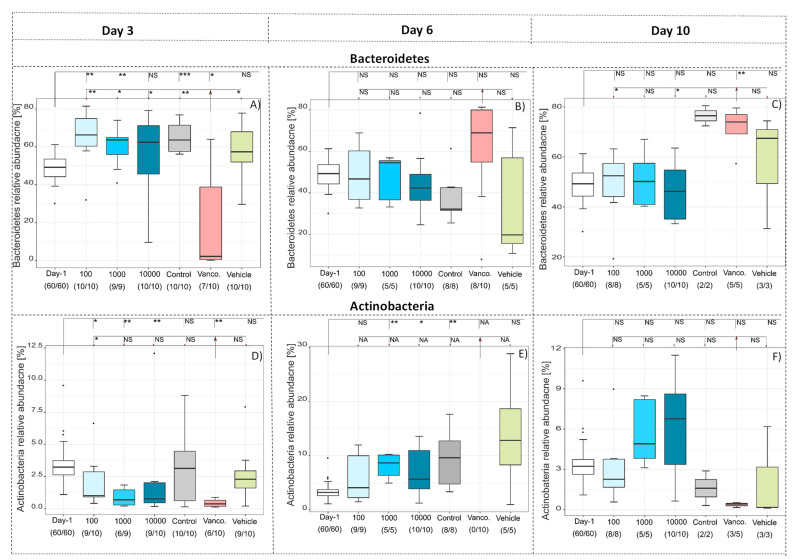
Relative abundance at phylum level of Bacteroidetes (**A**–**C**) and Actinobacteria (**A**–**E**) after 3 (**A**,**D**), 6 (**B**,**E**) and 10 (**C**,**F**) days at indicated treatment compared to the relative abundance before treatment (day -1). Significance compared to before treatment and Vancomycin values are *p* < 0.05 = *, *p* < 0.01 = **, *p* < 0.001 = ***, NS = not significant. Significant differences between the groups are marked by small letters.

**Figure 4:**
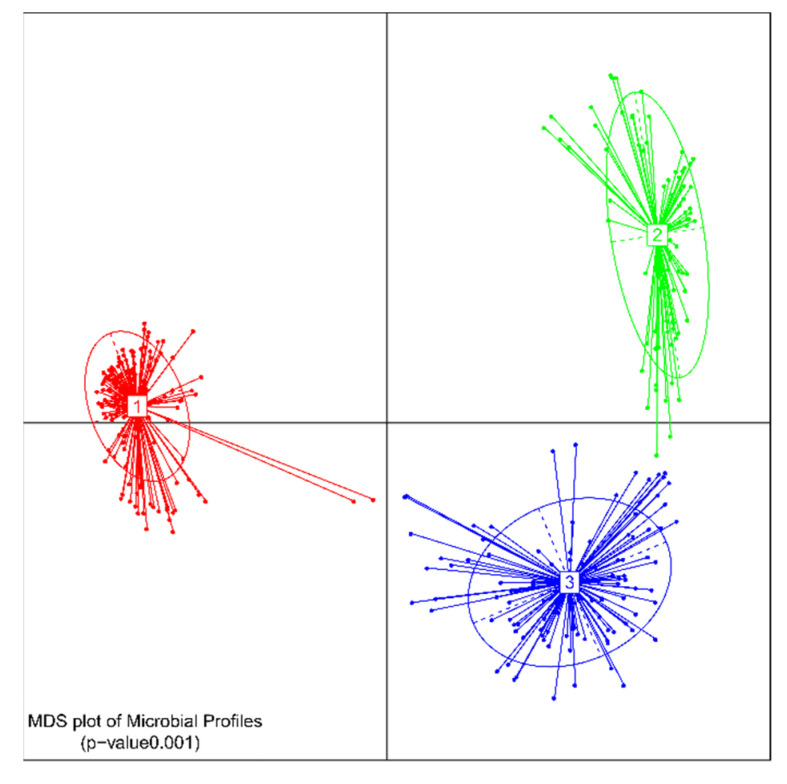
De novo clustering of all samples. Cluster 1 (red) shows the intestinal community of the hamsters before treatment, cluster 2 all treatment groups on day 3 as well as the Vancomycin-treated group throughout the study and cluster 3, all treatment groups from day 6 except the Vancomycin-treated group.

Looking at taxonomic differences, we focused on changes on phyla level (Figure 2 and Figure 3). All levels of significance compared to pretreatment and compared to the Vancomycin treated group are marked on the graph. It shows that the relative abundance of Firmicutes (*p* < 0.001,Figure 2A) and Actinobacteria (*p* < 0.05, Figure 3D) was significantly lower in all groups by day 3 due to the susceptibility to the antibody Clindamycin, whereas the relative abundance of Proteobacteria (*p* < 0.001, Figure 2A) and Bacteroidetes (*p* < 0.05, Figure 3A) increased significantly), except for the Vancomycin-treated group where Firmicutes, Actinobacteria and Bacteroidetes were significantly eliminated, indicating susceptibility to the antibiotic. In contrast, the relative frequency of Proteobacteria in the Vancomycin-treated group increased from hardly detectable to over 85%, which is consistent with the result reported by [12]. In the three groups treated with different concentrations of antibodies after day 6 and 10, the frequency of Bacteroidetes was similar (Figure 3B,C). Firmicutes were less frequent (Figure 2B,C), while Proteobacteria (Figure 2E,F) and Actinobacteria (Figure 3E,F) were significantly more frequent compared to before treatment The change of the microbiota can also be seen in Figure 4, which shows a de novo clustering of all samples based on the distance of their microbial profile. Cluster 1 (red) shows the intestinal community of the hamsters before treatment. The application of Clindamycin changed the intestinal microbiome, and the microbiome of the hamsters of all treatment groups differed on day three resulting in a new cluster (green) (*p* < 0.001). However, the microbiome of all treatment groups changed again until day six (blue cluster) (*p* = 0.001). The only exception was the group treated with antibiotics, where the microbial distance of the bacteria changed so little that they were assigned to group two over the entire study period. This means that although the diversity was equally diverse from day six onwards compared to before treatment, the relative composition changed in relation to the abundance. In the future, it should be investigated whether there was no change in the microbiome without the susceptibility antibiotic or whether the administration of milk proteins led to a change due to the actual treatment.

### 2.2. Over-Time Comparison of Treatment Groups

**Figure 5:**
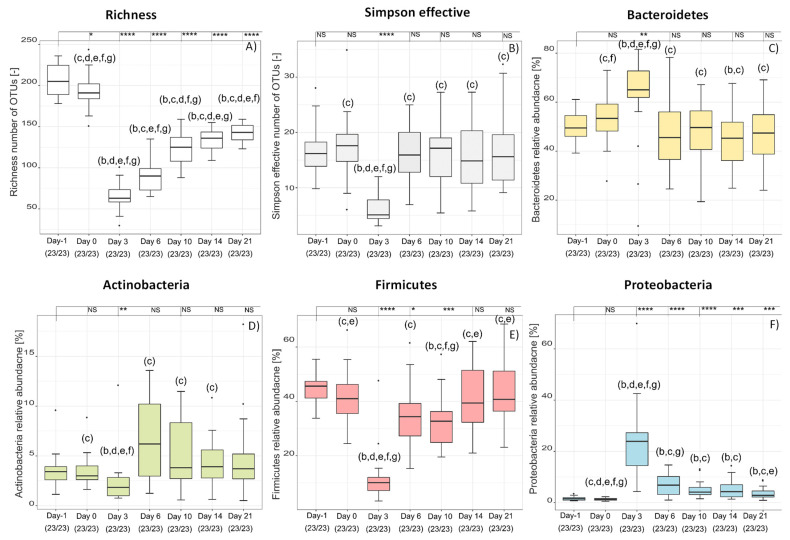
Richness (**A**), Simpson effective index (**B**) and relative abundance at phylum level of Bacteroidetes (**C**), Actinobacteria (**D**), Firmicutes (**E**), Proteobacteria (**F**) of the 23 hamster that survived due to the treatment with anti-CD-WPI (WPI 100, 1000, 10,000). Significance values are *p* < 0.05 = *, *p* < 0.01 = **, *p* < 0.001 = ***, *p* < 0.001 = ****, NS = not significant. Given significance values are in comparison to before treatment, significant differences between the groups are marked by small letters.

Interindividual changes over time and between groups were analyzed as well. There was no significant difference in the number of surviving animals (23/30) within the three groups (WPI 100, 1000, 10,000) in terms of number of species present, overall diversity or relative composition. Therefore, they were combined and fused into one group (Figure 5). At the end of treatment, the relative abundance of Firmicutes, Bacteroidetes and Actinobacteria (Figure 5C–E) was again at the same level as before treatment, while the relative abundance of Proteobacteria (Figure 5F) was still significantly (*p* < 0.001) increased.

### 2.3. Expanding the Level of Understanding of the Role of the Microbiome in CDI

The commensal gut microbiota is a complex community of microorganisms that exist in the gastrointestinal tract, consisting of about 300 ± 150 different species in humans [13]. The balance of this microecosystem is essential for the homeostasis of the host. It protects the intestine by providing colonization stability and resistance against the infection by pathogens [14]. Mechanisms involved in this protection are direct inhibition of *C. difficile* through bacteriocins [15] or indirect by bacteria-derived metabolites, nutrient depletion [16] or stimulation of host immune defenses [17]. Even though several studies on the role of the microbiome in CDI have been carried out, there are still many gaps in knowledge regarding to the role of the microbiome in CDI [2,14]

**Figure 6:**
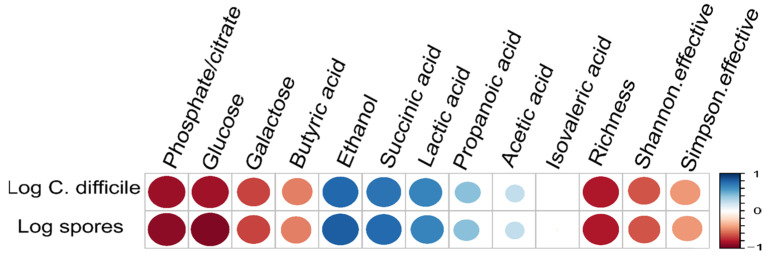
Heat map of pairwise correlation of *C. difficile* cell and spore counts (data from 5) with the metabolites phosphate/citrate, glucose, galactose, butyric acid, ethanol, succinate, lactic acid, propionic acid and isovaleric acid, and the alpha diversity indicators richness, Shannon effective index and Simpson effective index.

**Figure 7:**
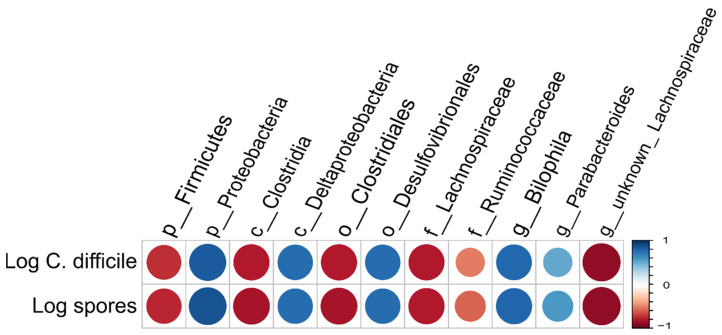
Simplified heatmap on different taxonomic levels where there is a significant correlation (>/< ±0.7) with *C. difficile*.

Therefore, irrespective of the therapy used, the linearized cell and spore numbers of *C. difficile* [5] were correlated with the different metabolites measured by RP-HPLC (Table A1) in intestinal fluid (Figure 6) and with the relative abundance of different species (Figure 7). *C. difficile* was significantly positively (correlation coefficient >0.5) correlated with the metabolites ethanol, succinate and lactic acid, and significantly negatively (correlation coefficient <−0.5) correlated with the metabolites phosphate/citrate (no differentiation possible with the used RP-HPLC method), glucose, galactose, butyric acid and alpha diversity indicators, richness and Simpson effective. In addition to the Simpson effective index, the Shannon effective index was also considered in this evaluation. In analogy to the Simpson effective index, the Shannon effective index is a measure of the equality of microbiome diversity, with a slightly different weighting on the abundance of the species present. Since both parameters are used in the literature, both are shown here. The individual pairwise correlation coefficients and *p*-values are shown in Table A2 and for some selected values in the body text in brackets.

**Figure 8:**
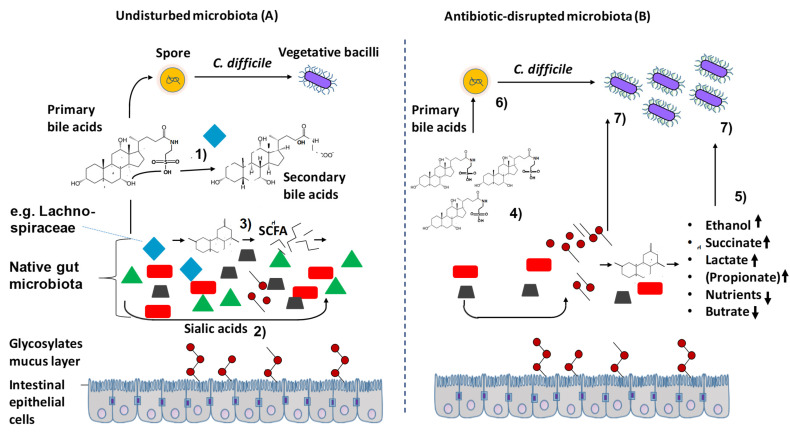
The role of the indigenous gut microbiota on CDI. Microbiota-mediated defense with native microbiota (**A**), omission of defense mechanism after antibiotic caused disruption of microbiota (**B**), modified from [17] and supplemented with our own correlation data.

The role of the indigenous intestinal microbiota for the CDI, which has been extended by our findings, is shown in Figure 8, hence the individual steps or processes are numbered. Although the exact mechanism by which a healthy microbiota suppresses the growth of *C. difficile* is not known, it is known that bile acids play an important role. The intact microbiota converts the primary bile acids into secondary bile acids (step 1 & 6), thereby inhibiting vegetative *C. difficile* by detergent-induced toxicity (Ridlon and Hylemon 2012). Correlation values of *C. difficile* spores/cells are given in brackets and the significance levels presented in Table A1 It was shown that at the taxonomic family level (−0.79/0.8) and genus level (−0.89/0.89), Lachnospiraceae correlated significantly negatively with C. difficile cell and spore counts (Figure 6 and Figure 7). This is consistent with findings of Reeves et al. (2012) with experiments using aseptic mice, where it was shown that administration of a single bacterium of the family Lachnospiraceae could reduce the cell density of *C. difficile*. Lachnospiraceae can convert primary bile acids into secondary bile acids and thus inhibit the growth of *C. difficile* (Figure 8A, step 1). In addition, Lachnospiraceae can produce butyric acid, which is also negatively correlated with the growth of *C. difficile*.

In addition, indigenous intestinal bacteria express sialidases that cleave sugars from glycosylated proteins bound to epithelial cells, which, in turn, release free sialic acid into the intestinal lumen [18] (Figure 8A, step 2). In addition, carbohydrates enter the colon via food, and fermenting gut bacteria break down the split carbohydrates and convert them into short chain fatty acids (SCFAs) [19] (Figure 8A, step 3). Succinate is a typical SCFA [16]. Among others, the metabolites glucose, galactose, sialic acid and succinate are used as energy sources for other commensal gut bacteria.

However, treatment with antibiotics, especially broad-spectrum antibiotics, significantly changes the intestinal microbiota and inactivates, for example, Lachnospiraceae, as shown by our results. The altered relative composition of the intestinal microbiome led to higher concentrations of metabolites, which correlate significantly positive with *C. difficile* in the case of sialic acid [16] succinate (r = 0.77) lactate (r = 0.67), ethanol (r = 0.82) and propionate (r = 0.41) (Figure 8B, step 5). This was likely due to the lack of organisms breaking these molecules down or because of the increased growth of organisms producing these metabolites. This indicates that a more hospitable environment for the growth of *C. difficile* is produced by these metabolites, as was also shown for succinate [20]. In addition, the dietary restriction, i.e., a low concentration of glucose (r = −0.92) and galactose (r = −0.68 favors the growth of *C. difficile* (Figure 8B, step 5). *C. difficile* can metabolize these molecules (Figure 8B step 7), followed by toxin production and CDI symptoms.

Our results contribute to a better understanding of the role of intestinal microbiome in CDI by correlating different metabolic by-products with *C. difficile* cell and spore counts. High concentrations of nutrients such as glucose and galactose, as well as butyric acid, inhibit the growth of *C. difficile*, whereas high concentrations of ethanol, lactic acid and succinate support the growth of *C. difficile*. The results also confirm that the specific approach using bovine antibodies does not interfere with the natural microbial balance and, therefore, is a sustainable alternative to the antibiotics used so far. As a follow-up, it is suggested that this knowledge could possibly be used to support the selection and cultivation of a so-called minimal consortium of intestinal bacteria, which may in the future be an alternative to FMT and would ideally complement our specific approach for CDI treatment.

## 3. Materials and Methods

### 3.1. Study Design

The study design is described in detail by Heidebrecht et al. (2019) [5]. Briefly, anti-*Clostridium difficile* whey protein isolate (anti-CD-WPI) powder was prepared from the milk or colostrum of specifically vaccinated cows. Cows were multiple vaccinated with inactivated TcdA and TcdB (accountable for CDI pathogenesis) and *C. difficile* cell/spore material to induce antigen-specific secretory immunoglobulin A (sIgA) and IgG antibodies in the milk (animal study approval number AZ 55.2-1-54-2532.6-17-12). Six groups of ten healthy hamsters each were infected orally with 100 spores of *C. difficile* strain 630 (tgcBiomics, Bingen, Germany) one day after the susceptibility antibody Clindamycin was provided. Three different concentrations were used based on their neutralization capacity (NC) against toxin A (anti-CD-WPI 10,000, anti-CD-WPI 1000, anti-CD-WPI 100) as described by [5]. Reference groups were treated with control WPI from the same cows before immunization, the standard of care antibiotic Vancomycin and liquidation buffer only. Hamsters were dosed with 1 mL WPI-solution/100 g body weight by oral gavage. Preparations were administered 3 h before and 3 h after bacterial challenge and then every 8 h during the consecutive days for 75 h in total. All procedures were conducted in accordance with the approved protocol of the ‘Institutional Animal Care and Use Committee’ (IACUC-2016-0015).

### 3.2. Sample Collection

A single fecal pellet was collected from individual cages of all hamsters. Samples were collected on day one, i.e., after five days of acclimation and before administration of Clindamycin, on day zero, i.e., after administration of Clindamycin and before first treatment, and thereafter on day 3, 6, 10, 14 and 21 from surviving hamsters. In addition, about 10 mL of caecal fluid was taken from all 60 hamsters either on the day of death due to the disease or at the study end (day 21) after euthanization of surviving hamsters. Samples were stored and shipped on dried ice and subsequently stored at −20 °C for further analysis.

### 3.3. Microbiome Analysis by Sequencing of 16S rRNA

Microbiome analysis was done as described by [21]. Paired end sequencing was performed by using specific primer (341 forward and 785 reverse) targeting the V3–V4 region of the hypervariable region of the 16S rRNA gene [22,23]. Amplicons were generated by a two-step (25 cycles) polymerase chain reaction (PCR), purified and pooled. The DNA quality and quantity of all samples were in the calibration range and were checked by water samples. The 2nM PCR-fragment was sequenced on a MiSeq (Illumina) at the Technical University of Munich (TUM) Microbiome Core Facility. The negative controls’ sole DNA stabilization buffer and water on every 96-well plate were inconspicuous and removed for further data processing.

### 3.4. Data Processing

Raw FASTQ files were demultiplexed using the pipeline ‘Integrated Microbial Next Generation Sequencing’ (IMNGS) [24], which is based on UPARSE approach [25]. Two errors in barcode sequences were the maximal allowed number. Reads were trimmed to the position of the first base with a quality factor <3 and then paired. The resulting sequences were size filtered, except those of assembled size <300 and >600 nucleotides. Paired reads with expected error >3 were further filtered out and the remaining reads were trimmed at each end by 10 nucleotides to prevent analysis of the distorted base composition regions at the beginning of the sequences. Operational taxonomic units (OTUs) were grouped with a sequence similarity of 97%, keeping only those with a relative abundance of >0.25% in at least one sample of the 412 samples (352 fecal samples and 60 ceacal samples). OTU tables of all study groups are provided in the Appendix A. The Rhea pipeline [26], a set of scripts of the statistical computing software R, was used for data processing. In brief, sequences were normalized to the minimum count of sequences observed. Samples with less than 2500 reads counts were excluded [26]. Microbial diversity between groups was calculated by generalized Unifrac distances [27]. The Ribosomal Database Project (RDP v.9) classifier (Wang et al., 2007) was used to assign taxonomies at 80% confidence level. Important unidentified OTUs were classified using ExTaxon. *p* values were corrected for multiple comparisons according to the Benjamini-Hochberg method. Only taxa with a prevalence of 10% (proportion of samples positive for the given taxa) in at least one group were considered for statistical testing.

### 3.5. Determination of Intestinal Metabolites with Reversed-Phase High-Performance Liquid Chromatography (RP-HPLC)

A new HPLC method was established to measure the different metabolites. The intestinal fluid was sterile-filtered directly into a vial using a 0.2 µm syringe filter. An Aminex HPX-87H 300 × 7.8 mm (Bio-Rad, Hercules, CA, USA) and a guard column were used for separation, in which 20 µL were injected and isocratically eluted with 0.005 M sulfuric acid at a flow rate of 0.450 mL/min and a temperature of 35 °C. The detection was done by measuring refractive index. With this method the metabolites glucose, galactose, succinic acid, lactic acid, acetic acid, propionic acid, ethanol, butyric acid and iso-valeric acid could be quantified simultaneously. Phosphate and citrate elute at the same time with this method and therefore could not be differentiated.

## 4. Conclusions

From the comparison of the groups among themselves and with the group treated with Vancomycin and integrating our previous data (8) published in Heidebrecht et al. (2019), several conclusions can be drawn. As described above, an active and antibody-induced mechanism must take place in the intestine which causes the survival of the animals. Our antibodies can inactivate foreign *C. difficile* antigens through various mechanisms of action. These include opsonization, activation of the complement system, agglutination, prevention of adhesion and direct neutralization. It is unlikely, or at least not the primary mechanism of action, that cell adhesion is prevented, and direct neutralization of the *C. difficile* cells occurs. Had this been the case, there would not have been initial spore germination and cell growth [5], but the cells would have been directly eliminated. The later decline in cell growth between days six and eight indicates that the host’s own immune system was activated (which is a delayed process), either by activation of the complement system or by direct labelling of the cells for the host’s own defense cells. However, since cell decline was also observed in some control groups (e.g., control WPI), the deduced conclusion is that both indirect regeneration of intestinal microbial diversity (as observed in all groups except the antibiotics group) and direct antibody-induced inactivation were crucial for cell and spore decline of *C. difficile.* In other words, prevention of recurrences (only observed in the groups treated with active antibodies) can only be achieved if both mechanisms are given the chance to work, which is the case for Ig-based CDI treatment instead of antibiotics.

## Data Availability

The data is available in this manuscript.

## Appendix A

**Table A1:** Concentration of different metabolites in intestinal fluid measured by RP-HPLC.

	Phosphate [mg/mL]	Glucose [mg/mL]	Galactose [mg/mL]	Succinic Acid [mg/mL]	Lactic Acid [mg/mL]	Acetic Acid [mg/mL]	Propionic Acid [mg/mL]	Ethanol [mg/mL]	Butyric Acid [mg/mL]	Iso-Valeric Acid [mg/mL]
Placebo	2.68	1.23	0.23	0.09	0.34	1.77	0.42	0.18	0.40	0.60
	1.44	0.00	0.21	2.05	0.25	3.90	1.15	0.76	0.25	0.70
	0.96	0.00	0.02	0.84	1.30	1.11	0.29	1.66	0.48	0.77
	1.36	0.00	0.13	2.01	0.97	2.37	1.11	1.43	0.24	0.50
	1.03	0.00	0.09	2.29	0.74	2.51	1.23	2.10	0.52	0.16
	1.06	0.00	0.18	1.37	1.10	2.03	0.96	3.57	0.48	0.14
	1.23	0.00	0.04	1.48	0.13	3.83	1.25	2.28	0.49	0.24
	1.54	0.24	0.24	1.87	1.53	4.04	1.18	1.89	0.82	0.32
WPI 1000	3.28	2.89	0.27	0.11	0.11	2.25	0.31	0.45	1.50	0.03
	3.29	1.45	0.31	0.04	0.13	1.29	0.20	0.08	0.63	0.02
	2.84	1.74	0.27	0.03	0.16	1.29	0.17	0.05	0.90	0.70
	3.25	1.87	0.26	0.79	0.14	1.86	0.29	0.00	1.06	0.46
	1.60	0.67	0.16	0.03	0.13	0.76	0.11	0.02	0.49	0.01
WPI100	2.83	1.08	0.36	0.09	0.19	1.68	0.27	0.03	0.81	0.53
	3.91	2.88	0.44	0.11	0.31	3.02	0.27	0.07	2.04	1.02
	1.02	0.04	0.11	0.19	0.25	0.64	0.03	0.96	0.28	0.02
	2.91	1.37	0.19	0.01	0.29	1.53	0.15	0.10	1.03	0.49
	3.06	1.47	0.23	0.04	0.18	1.66	0.18	0.03	1.05	0.39
	3.04	1.85	0.28	0.22	0.15	1.58	0.42	0.03	0.84	0.82
	3.34	1.98	0.32	0.05	0.09	1.86	0.27	0.28	1.05	0.61
	2.45	1.28	0.18	0.03	0.21	1.11	0.30	0.01	0.61	0.64
	3.08	1.52	0.51	0.05	0.12	1.60	0.31	0.06	0.99	0.53
WPI10000	3.00	1.85	0.29	0.27	0.31	1.73	0.27	0.12	1.12	0.04
	3.02	1.44	0.59	0.02	0.10	1.68	0.20	0.07	1.00	0.04
	3.17	2.30	0.25	0.09	0.08	1.62	0.17	0.10	1.10	0.46
	3.06	1.76	0.18	0.01	0.09	1.09	0.12	0.01	0.77	0.44
	2.96	1.78	0.20	0.17	0.07	1.61	0.57	0.00	0.86	0.04
	3.08	1.64	0.27	0.05	0.13	1.34	0.25	0.09	0.75	0.62
	2.91	1.09	0.43	0.48	0.08	1.33	0.21	0.12	0.86	0.76
	2.81	1.80	0.28	0.04	0.11	1.44	0.15	0.02	0.89	0.44
	3.22	1.79	0.36	0.10	0.07	1.31	0.19	0.05	0.86	0.58
	3.48	2.00	0.29	0.05	0.16	1.51	0.20	0.04	1.25	0.61
Vancomycin	1.73	0.00	0.14	0.82	0.80	2.56	1.09	0.64	1.21	0.33
	1.67	0.00	0.06	1.36	0.41	2.03	0.26	1.07	0.98	0.33
	1.24	0.07	0.08	0.84	0.19	2.59	0.94	5.71	0.97	0.24
	2.61	0.77	0.20	0.03	0.22	0.84	0.13	0.14	0.63	0.02
	1.62	0.00	0.09	1.35	0.77	3.28	1.05	0.34	1.03	0.28
Vehicle	1.02	0.00	0.09	0.43	0.44	0.71	0.09	0.25	0.43	0.58
	0.90	0.00	0.13	0.89	0.00	1.39	0.47	1.88	0.35	0.07
	1.04	0.00	0.18	0.62	0.21	1.26	0.35	1.70	0.58	0.62
	0.80	0.10	0.07	0.13	0.20	1.20	0.07	0.74	0.44	0.04
	0.97	0.06	0.14	0.38	0.07	0.63	0.23	0.09	0.23	0.04
	1.28	0.15	0.23	1.41	0.25	3.34	1.30	1.92	1.07	0.24

**Table A2:** Individual pairwise correlation coefficients and *p*-values.

Variable 1	Variable 1	Correlation	*p*-Value	Number of Supporting Data
Phosphate/Citrate	Log value of vegetative *C. difficile* count	−0.86	2.9 × 10^−13^	43
Phosphate/Citrate	Log value of *C. difficile* spore count	−0.89	8.88 × 10^−16^	43
Glucose	Log value of vegetative *C. difficile* count	0.85	1.67 × 10^−09^	31
Glucose	Log value of *C. difficile* spore count	−0.92	1.05 × 10^−13^	31
Galactose	Log value of vegetative *C. difficile* count	−0.67	8.79 × 10^−7^	43
Galactose	Log value of *C. difficile* spore count	−0.68	5.89 × 10^−7^	43
Butyric acid	Log value of vegetative *C. difficile* count	−0.54	1.78 × 10^−4^	43
Butyric acid	Log value of *C. difficile* spore count	−0.51	4.83 × 10^−4^	43
Ethanol	Log value of vegetative *C. difficile* count	0.79	9.44 × 10^−10^	41
Ethanol	Log value of *C. difficile* spore count	0.82	4.03 × 10^−11^	41
Succinic acid	Log value of vegetative *C. difficile* count	0.74	1.61 × 10^−8^	43
Succinic acid	Log value of *C. difficile* spore count	0.77	1.11 × 10^−9^	43
Lactic acid	Log value of vegetative *C. difficile* count	0.61	2.10 × 10^−5^	42
Lactic acid	Log value of *C. difficile* spore count	0.67	1.24 × 10^−6^	42
Richness	Log value of vegetative *C. difficile* count	−0.80	9.33 × 10^−15^	60
Richness	Log value of *C. difficile* spore count	−0.81	5.55 × 10^−15^	60
Shannon effective Index	Log value of vegetative *C. difficile* count	−0.61	2.22 × 10^−7^	60
Shannon effective Index	Log value of *C. difficile* spore count	−0.63	8.06 × 10^−8^	60
Simpson effective Index	Log value of vegetative *C. difficile* count	−0.41	1.04 × 10^−3^	60
Simpson effective Index	Log value of *C. difficile* spore count	−0.44	4.86 × 10^−4^	60
Simpson effective Index	Log value of *C. difficile* spore count	−0.80	9.33 × 10^−15^	60
